# A comparative and ontogenetic examination of mitochondrial function in Antarctic notothenioid species

**DOI:** 10.1007/s00360-022-01461-6

**Published:** 2022-09-14

**Authors:** Milica Mandic, Amanda J. Frazier, Andrew W. Naslund, Anne E. Todgham

**Affiliations:** Department of Animal Science, University of California Davis, 2251 Meyer Hall, Davis, CA 95616 USA

**Keywords:** Mitochondrial function, Oxidative phosphorylation, Notothenioids, Ontogeny, Metabolic capacity, Permeabilized fibers

## Abstract

Notothenioidei fishes have evolved under stable cold temperatures; however, ocean conditions are changing globally, with polar regions poised to experience the greatest changes in environmental factors, such as warming. These stressors have the potential to dramatically affect energetic demands, and the persistence of the notothenioids will be dependent on metabolic capacity, or the ability to match energy supply with energy demand, to restore homeostasis in the face of changing climate conditions. In this study we examined aerobic metabolic capacity in three species, *Trematomus bernacchii*, *T. pennellii* and *T. newnesi*, and between two life stages, juvenile and adult, by assessing mitochondrial function of permeabilized cardiac fibers. Respiratory capacity differed among the adult notothenioids in this study, with greater oxidative phosphorylation (OXPHOS) respiration in the pelagic *T. newnesi* than the benthic *T. bernacchii* and *T. pennellii*. The variation in mitochondrial respiratory capacity was likely driven by differences in the mitochondrial content, as measured by citrate synthase activity, which was the highest in *T. newnesi*. In addition to high OXPHOS, *T. newnesi* exhibited lower LEAK respiration, resulting in greater mitochondrial efficiency than either *T. bernacchii* or *T. pennellii*. Life stage largely had an effect on mitochondrial efficiency and excess complex IV capacity, but there were little differences in OXPHOS respiration and electron transfer capacity, pointing to a lack of significant differences in the metabolic capacity between juveniles and adults. Overall, these results demonstrate species-specific differences in cardiac metabolic capacity, which may influence the acclimation potential of notothenioid fishes to changing environmental conditions.

## Introduction

A climate shift coincident with a significant decrease in atmospheric carbon dioxide (CO_2_) at the end of the Eocene Epoch, approximately 34 million years ago, triggered the formation of terrestrial and sea glaciation in the Antarctic (Tripati et al. 2005). As the Antarctic Circumpolar Current formed, this created physical isolation of the fish fauna to the Antarctic waters, paving the way for evolution in response to the coldest temperatures in the world. Antarctic notothenioid fishes for the past several million years have lived under stable thermal regimes and currently experience narrow seasonal fluctuations in temperatures ranging from − 1.8 to − 0.5 °C at the Southern Ocean’s highest latitudes (McMurdo Sound, Ross Sea; Hunt et al. 2003). It has been suggested that the radiation of the Antarctic fishes in the cold environment was associated with key cellular adaptations as well as with evolutionary loss of characters and function with subsequent adaptive recovery (Montgomery and Clements 2000). To survive and thrive in this environment, Antarctic fishes have evolved antifreeze proteins (DeVries and Wohlschlag 1969), loss of hemoglobin and myoglobin expression in Family Channichthyidae (icefishes) that was subsequently compensated by enlarged hearts and large blood volume (see review O’Brien 2016), loss of the classic inducible heat shock protein response (Hofmann et al. 2000), large diameter of fast and slow muscle fibers (Dunn et al. 1989) and high mitochondrial densities (Archer and Johnston 1991). These traits provided the necessary resilience of the species to sub-zero Antarctic waters, but at a potential cost of decreased ability to acclimate to shifting environmental conditions (Beers and Jayasundara 2015; Todgham and Mandic 2020).

Climate change is expected to have profound impacts on the physiochemical properties of waters surrounding Antarctica. The Southern Ocean has experienced some of the fastest changes in temperature in the world (Vaughan et al. 2003) and ocean temperature is projected to increase by 1–3 °C by 2100 (Pörtner et al. 2019). Simultaneously, open ocean seawater pH may decrease by 0.3–0.5 units (IPCC, 2013; McNeil et al. 2010), and the duration of seasonal low pH may increase in near-shore sites of McMurdo Sound (Kapsenberg et al. 2015). Warming has altered the dynamics of glacial melt impacting salinity (Dierssen et al. 2002), and dissolved oxygen (O_2_) has been on a decline in the world’s oceans (Schmidtko et al. 2017). Antarctic fishes will face a combination of multiple stressors and the extent of their vulnerability to climate change will likely be determined by metabolic capacity, or the ability to match energy supply with increased energy demand in response to environmental change (Todgham and Mandic 2020). The degree of impact will vary among species, depending on how a species’ energy metabolism was shaped by factors such as the historic trajectory of character and function evolution. The icefish, *Chaenocephalus aceratus*, for example, lack hemoglobin and were found to have a lower aerobic metabolic capacity than the red-blooded *Notothenia coriiceps*, contributing, at least in part, to lower thermal tolerance (O’Brien et al. 2018). Projected ocean warming, therefore, may exert a greater negative effect on the icefishes than the red-blooded notothenioids, demonstrating that despite the general stenothermy of Antarctic fishes (Somero and DeVries 1967), certain species may be better poised to cope with the changing environmental conditions.

Variation in species metabolic capacity can also be influenced by differences in ecological niches and activity levels (Teulier et al. 2019). Notothenioids are derived from benthic ancestors and while the majority remained benthic, some species have become secondarily pelagic through retention of larval characteristics (Eastman 1993; Montgomery and Clements, 2000). The pelagic species, *Lepidonotothen nudifrons* and *Trematomus newnesi*, with moderately active to active lifestyles, respectively, were found to have greater oxidative capacities of isolated mitochondria than the *N. coriiceps*, a more sedentary, benthic species (Johnston et al. 1998). Likewise, an examination of isolated mitochondria between benthic notothenioids and the pelagic *Pleuragramma antarcticum* determined that the benthic species had 50% lower rates of oxidative phosphorylation respiration of the mitochondria (Martinez et al. 2013). Evidence from the two studies suggests that oxidative phosphorylation, and hence aerobic metabolism capacity, of pelagic notothenioids is greater than that of the benthic species. While it is unknown how these metabolic differences will impact the acclimation response and ultimately whole animal performance, understanding the metabolic capacity of benthic versus pelagic Antarctic fishes is an important step in characterizing the metabolic potential of the species in face of changing ocean conditions (Todgham and Mandic 2020).

Examining the role of mitochondrial function in acclimation capacity of Antarctic species to multiple stressors (temperature, *P*CO_2_, salinity, hypoxia) over extended period of time (e.g. months to years) is logistically challenging in remote environments such as the Antarctic. An alternative to lab manipulated experiments is to monitor changes in mitochondrial trait function over time in field-caught fishes to determine if there is a shift in metabolic capacity to compensate for changing environmental conditions in the field. For this approach, the critical initial step is to assess current mitochondrial performance of species, by conducting baseline measurements of mitochondrial function in a comparative framework. In this study, we examined mitochondrial performance in permeabilized cardiac fibers and determined function through each component of the electron transport system (ETS) of three species of adult Antarctic fishes, *T. bernacchii, T. pennellii, and T. newnesi*, found at high latitudes of McMurdo Sound, Ross Sea and occupying two different ecological niches, the benthic (*T. bernacchii* and *T. pennellii*) and the pelagic (*T. newnesi*). These experiments provide detailed measurements of baseline mitochondrial performance of adult stages of the three species. As early life stages may be particularly vulnerable to environmental stressors (Pörtner and Peck 2010; Pankhurst and Munday 2011), we additionally examined baseline cardiac mitochondrial performance of juvenile *T. bernacchii* and *T. pennellii*. While there is some evidence to suggest a negative impact of elevated temperature on growth in juvenile *T. bernacchii* (Sandersfeld et al. 2015) and elevated temperature and *P*CO_2_ on survival and development in embryonic dragonfish *Gymnadraco acuticeps* (Flynn et al. 2015), little is known about how the juvenile pelagic stage of notothenioids compares to that of the adult. The inclusion of an ontogenetic approach is vital in obtaining relevant information for meaningful foundational understanding of these species’ metabolic performance.

## Materials and methods

### Experimental animals

Antarctic notothenioid species, *Trematomus bernacchii*, *T*. *pennellii* and *T*. *newnesi*, belonging to subfamily Trematominae, were collected in October and November of 2019 in McMurdo Sound, Ross Sea, Antarctica. Juvenile and adult stages of *T. bernacchii* and *T. pennellli* species were captured at the Intake Jetty in front of McMurdo station (77° 51.072′ S, 166° 39.878′ E), while adult *T. newnesi* were captured at Cape Evans Ice Wall (77° 38.407′ S, 166° 31.068′ E). We have not been able to find locations in McMurdo Sound where we can capture *T. newnesi* juveniles, despite looking since 2013. Holes were drilled through the annual sea ice for scientific SCUBA divers to collect juvenile fish and for catching adult fish by hook-and-line fishing. Specimens were transported using well aerated, insulated coolers to Crary Science and Engineering Center at McMurdo station within 3 h of collection and maintained at − 1.5 °C in a flow through system. Transportation of fish from the Jetty to McMurdo Station was significantly shorter (~ 15 min) than from Cape Evans Ice Wall (~ 2.5 h). As such, specimens from the Jetty were allowed to recover for 24 h, while specimens from Cape Evans Ice Wall were allowed to recover for 48 h. Fish were not fed during the recovery period, following which they were euthanized by spinal severance. Heart ventricles were quickly dissected for immediate preparation of permeabilized muscle fibers or frozen in liquid N_2_ and stored at − 80 °C for later analysis. All procedures for animal use and experimentation were carried out in compliance with the University of California, Davis (Protocol #20558).

### Permeabilized muscle fiber respiration

Heart ventricles of three juvenile individuals or a sample of a heart ventricle of an adult fish (~ 5 mg) were immediately placed into a relaxing and preservation solution at pH 7.1 (in mM): 2.77 CaK_2_EGTA, 7.23 K_2_EGTA, 5.77 Na_2_ATP, 6.56 MgCl6H_2_O, 20 taurine, 15 creatine phosphate, 20 imidazole, 0.5 dithiothreitol (DTT), 50 MES hydrate. Muscle fibers were mechanically separated in relaxing and preservation solution using dissecting probes under a dissecting microscope. Fibers were then placed in the same solution containing saponin (50 μg ml^−1^) and gently mixed using an orbital shaker for 30 min to chemically permeabilize the fibers. Following permeabilization, the fibers were rinsed three times for 5 min in respiration medium (in mM unless otherwise indicated): 0.5 EGTA, 3 MgCl6H_2_O, 60 lactobionic acid, 20 taurine, 10 KH_2_PO_4_, 20 HEPES, 110 D-Sucrose, 1 g l^−1^ BSA. All steps were performed in a temperature-controlled room set at 0 °C.

Permeabilized ventricle fibers were quickly weighed and placed into fresh respiration medium in the high-resolution respirometer (Oxygraph-2 k, Oroboros Instruments, Innsbruck, Austria). Respiration rate of fibers was measured in continuously stirred 2 ml of respiration medium at 0.5 °C, the lowest stable temperature we reliably could achieve in the Oroboros chambers. The O_2_ concentration in the chambers during the trials were maintained above air saturation (> 400 μM at 0.5 °C) by injecting O_2_ into the gas phase above the respiratory medium to avoid O_2_ limitation to the mitochondria. An instrumental background O_2_ flux was measured in the same range of elevated O_2_ levels used in the experimental trials; the background corrections were accounted for in the data analysis. Oroboros was calibrated daily to 100% air saturation and weekly to both 100% and 0% air saturation.

Once in the chambers, fibers were allowed to rest for 25 min prior to the addition of pyruvate (5 mM) and malate (2 mM), which stimulated NADH-linked substrate LEAK-I respiration (*L*_N_). Step-wise additions of ADP to saturating levels (4 mM) caused NADH-linked substrate OXPHOS-I respiration (*P*_PM_), after which succinate (10 mM) was added to achieve NADH and succinate-linked substrate OXPHOS-I,II respiration (*P*_PMS_). The integrity of the outer mitochondrial membrane was assessed with the addition of cytochrome *c* (10 μM); if cytochrome *c* stimulated respiration by 5% or more, the permeabilized fiber preparation was considered compromised and taken out of the analysis. Oligomycin (2.5 μM) was added to inhibit ATP synthase and determine oligomycin induced LEAK-I,II respiration (*L*_Omy_). This was followed by additions of carbonyl cyanide *p*-trifluoro-methoxyphenyl hydrazone (FCCP; 3 μM) until maximum stimulation to fully uncouple respiration, measuring electron transfer (ET) pathway capacity (*E*_PMS_). Rotenone (0.5 μM) was then added to inhibit complex I in order to determine complex II contribution to the electron transfer pathway capacity (*E*_S(Rot)_) and antimycin (2.5 μM) to inhibit complex III and, therefore, the remaining mitochondrial respiration rate, termed residual O_2_ consumption or ROX. However, with the exception of juvenile *T. pennellii*, mitochondrial respiration following addition of antimycin did not go below that of LEAK respiration. It is possible that our combination of inhibitors did not fully arrest mitochondrial respiration, therefore, data reported is for total rather than ROX-corrected respiration. Lastly, ascorbate (2 mM) and *N*,*N*,*N*,*N*-tetramethyl-*p*-phenylenediamine (TMPD; 0.5 mM) was added to maximally stimulate complex IV (*E*_TM_). Respiration rates were normalized to tissue wet mass. Respiratory control ratio (RCR), an estimate of mitochondrial efficiency, is a ratio of mitochondrial respiration used for ATP synthesis to the respiration required to offset the proton leak, and was calculated by taking the ratio of OXPHOS respiration to LEAK state (RCR-I: OXPHOS-I and LEAK-I; RCR-I,II: OXPHOS-I,II and LEAK-I,II). The limitation of OXPHOS by the phosphorylation pathway was calculated by the phosphorylation pathway control ratio of *P*_PMS_ to *E*_PMS_ and ratio of *E*_TM_ to *P*_PMS_ was calculated to determine the excess complex IV respiration relative to OXPHOS capacity. Sample size was 10 individuals for each species and life stage.

### Heart ventricle mitochondrial enzyme activity

Maximal activities of citrate synthase (CS) and cytochrome *c* oxidase (COX) were measured in the frozen ventricle aliquots. For adult fish, an aliquot was the remainder of the ventricle not destined for the permeabilized fiber experiment, while for juvenile fish, an aliquot comprised of ventricles of three individual fish. Aliquots were quickly weighed and homogenized in 10 volumes of ice-cold homogenizer buffer (100 mM KH_2_PO_4_, 1 mM EGTA, 1 mM EDTA, pH = 7.2) using the Bio-Gen PRO200 homogenizer (Pro Scientific Inc.). The homogenate was centrifuged at 1000 *g* for 10 min at 4 °C and the supernatant was collected and divided into separate aliquots for CS and COX assays.

Maximal enzyme activities were determined spectrophotometrically (Synergy HT, BioTek) by measuring the appearance of 5-thio-2-nitrobenzoic acid (TNB) at 412 nm for CS and by disappearance of ferrocytochrome c by its oxidation to ferricytochrome c at 550 nm for COX at 5 °C. Enzyme activity was assayed in the following conditions (in mM): CS [*ε* = 14.15 (mmol l^−1^)^−1^ cm^−1^] = 100 KH_2_PO_4_ at pH 8.0, 0.15 5,5’-dithiobis-2-nitrobenzoic acid, 0.3 acetyl-CoA, 0.5 oxaloacetate; COX [*ε* = 19.6 (mmol l^−1^)^− 1^ cm^−1^] = 100 KH_2_PO_4_ at pH 7.2, 0.2 reduced cytochrome c. Measured activities were assayed in triplicate for CS and quadruplicate for COX. Total protein of each homogenate sample was determined using the bicinchoninic acid method (Smith et al., 1985) with bovine serum albumin as the protein standard (Thermo Fisher Scientific). Sample size was 8 for adult *T. bernacchii*, *n* = 7 for adult *T. pennellii* and *n* = 9 for adult *T. newnesi* and 5 for juveniles of *T. bernacchii and T. pennellii* for CS activity. Sample size was 10 individuals for each species and life stage for COX activity.

### Statistical analysis

All statistical analyses were performed in R (v3.6.2, R Core Team 2019). For the adult data, the effect of species on aspects of mitochondrial function (LEAK, OXPHOS, ET capacity, RCR, *E*_TM_, phosphorylation pathway control ratio and ratio of *E*_TM_ to *E*_PMS_) was tested using one-way analysis of variance (ANOVA) in the ‘car’ package (Fox and Weisberg 2011). For the juvenile and adult comparison, the effects of species and life history stage on mitochondrial function were tested using two-way ANOVA. Data were tested for normality (Shapiro–Wilk test), equal variance (Levene’s test) and visually inspected for linearity, normality, homoscedasticity and identification of influential cases using the ‘stats’ package (R Core Team 2019) and the ‘car’ package (Fox and Weisberg, 2011). If the data failed these tests, they were transformed. Tukey’s post hoc test was performed if significant difference was detected using either the one-way or two-way ANOVA. Adult mitochondrial data were analyzed twice for the among-adult comparison for the three species and for the adult versus juvenile comparison for *T. bernacchii* and *T. pennellii*. As such, P-values were bonferroni corrected to account for a potentially inflated Type I error. Significance was set at *P* < 0.05.

## Results

### Mitochondrial respiration in adult notothenioids

**Fig. 1:**
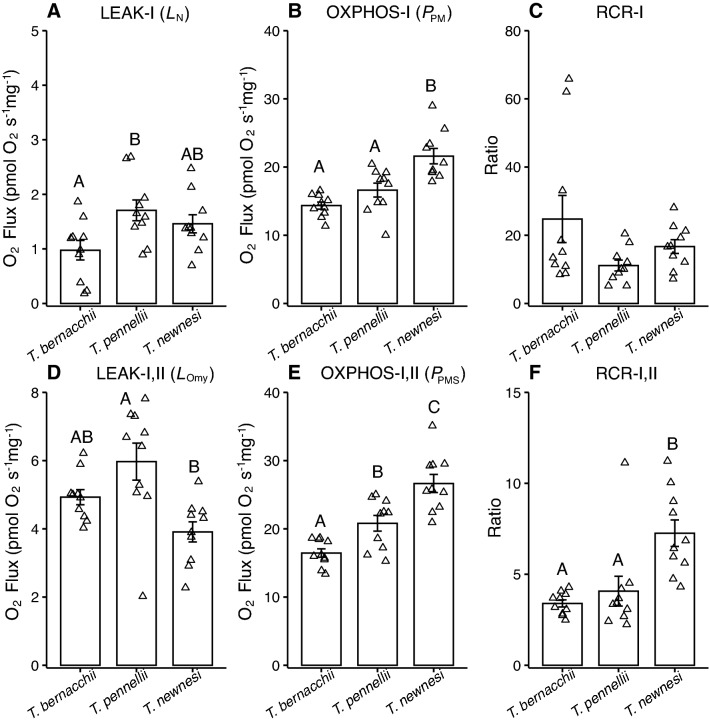
Mitochondrial respiration during LEAK and OXPHOS states in permeabilized ventricle fibers of adult *Trematomus bernacchii*, *T. pennellii* and *T. newnesi*. There was a significant effect of species on **A** LEAK-I respiration (*L*_N_; ANOVA; *F*_(2,27)_ = 4.3, *P* = 0.04) and **B** OXPHOS-I respiration measured with maximal ADP and substrates of complex I (*P*_PM_; ANOVA; *F*_(2,27)_ = 16.1, *P* < 0.01). There was no effect of species on **C** respiratory control ratio RCR-I (*P*_PM_/*L*_N_; ANOVA; *F*_(2,27)_ = 3.0, *P* = 0.14). There was a significant effect of species on **D** LEAK-I,II respiration (*L*_Omy_; ANOVA; *F*_(2,27)_ = 7.4, *P* < 0.01), and **E** OXPHOS-I,II respiration measured with maximal ADP and substrates of complex I and II (*P*_PMS_; ANOVA; *F*_(2,27)_ = 22.5, *P* < 0.01), and **F** RCR-I,II (*P*_PMS_/*L*_Omy_; ANOVA; *F*_(2,27)_ = 14.0, *P* < 0.01). Sample size was 10 individuals for each species. Different letters indicate significant differences among species. Data are presented as mean ± SEM

Coupled state respiration, LEAK and OXPHOS, were assessed in the ventricles of three adult Antarctic notothenioids (Fig. 1). There was a significant effect of species on LEAK-I respiration through complex I (Fig. 1A) and oligomycin-inhibited LEAK-I,II respiration through complexes I and II (Fig. 1D). LEAK-I and LEAK-I,II respiration was the highest in *T. pennellii*, although not significantly so from *T. newnesi* for LEAK-I and *T. bernacchii* for LEAK-I,II; there were no differences in LEAK-I or LEAK-I,II respiration between *T. bernacchii* and *T. newnesi*. The respiratory capacity for OXPHOS was significantly higher in *T. newnesi* (Fig. 1B,E). Complex I fueled OXPHOS-I respiration was 1.5-fold higher in *T. newnesi* than either *T. bernacchii* or *T. pennellii* (Fig. 1B). Respiration rates increased with addition of succinate across all species, and complex I and II fueled OXPHOS-I,II respiration was 1.6-fold and 1.4-fold higher in *T. newnesi* than *T. bernacchii* or *T. pennellii,* respectively (Fig. 1E).

Respiratory control ratio (RCR), an estimate of mitochondrial coupling and contribution of LEAK respiration to OXPHOS capacity, ranged between 11.1 ± 1.6 in *T. pennellii* and 24.8 ± 6.9 in *T. bernacchii*, although this was not significant different between species (Fig. 1C). There was an effect of species on complex I and II RCR-I,II with the highest RCR-I,II in *T. newnesi*, driven by high OXPHOS-I,II respiration and low LEAK-I,II respiration (Fig. 1F).

**Fig. 2:**
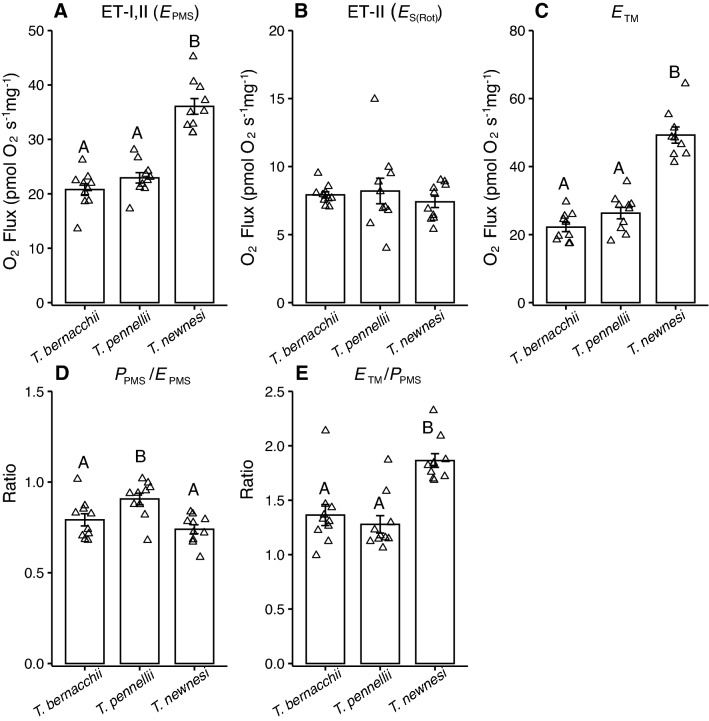
Electron transport capacity in ventricle fibers of adult *Trematomus bernacchii*, *T. pennellii* and *T. newnesi*. There was a significant effect of species on **A** electron transport capacity (ET-I,II) measured in mitochondria uncoupled with FCCP using substrates of complex I and II (*E*_PMS_: ANOVA; *F*_(2,27)_ = 49.6, *P* < 0.01). There was no effect of species on **B** ET-II through complex II (*E*_S(Rot)_; non-parametric Kruskal–Wallis ANOVA; *χ*^2^ = 0.6, *P* = 1). There was a significant effect of species on **C** complex IV respiration stimulated by TMPD and ascorbate (*E*_TM_; ANOVA; *F*_(2,27)_ = 62.9, *P* < 0.01), **D** the phosphorylation pathway control ratio (*P*_PMS_/*E*_PMS_; ANOVA; *F*_(2,27)_ = 7.9, *P* < 0.01) and **E** excess complex IV capacity calculated as the ratio of complex IV to OXPHOS respiration (*E*_TM_/*P*_PMS_; ANOVA: *F*_(2,27)_ = 15.9, *P* = 0.01). Sample size was 10 individuals for each species. Different letters indicate significant differences among species. Data are presented as mean ± SEM

Heart mitochondrial ET pathway capacity through complex I and II (*E*_PMS_) significantly differed among the species, with highest capacity in *T. newnesi* and no difference between *T. bernacchii* and *T. pennellii* (Fig. 2A). There was no difference among the species in ET pathway capacity through complex II only (*E*_S(Rot)_; Fig. 2B). Maximally stimulated complex IV respiration was significantly higher in *T. newnesi* than either *T. bernacchii* or *T. pennellii* (Fig. 2C) and *T. newnesi* had significantly higher excess complex IV capacity than either *T. bernacchii* or *T. pennellii* (Fig. 2E). There was a significant species effect on the phosphorylation pathway control ratio, with *T. pennellii* exhibiting the highest *P*_PM_ to *E*_PMS_ ratio (Fig. 2D). For all three species the ratio was below 1, indicating that OXPHOS respiration was not constrained by ET capacity.

### Mitochondrial function in juvenile vs. adult notothenioids

**Fig. 3:**
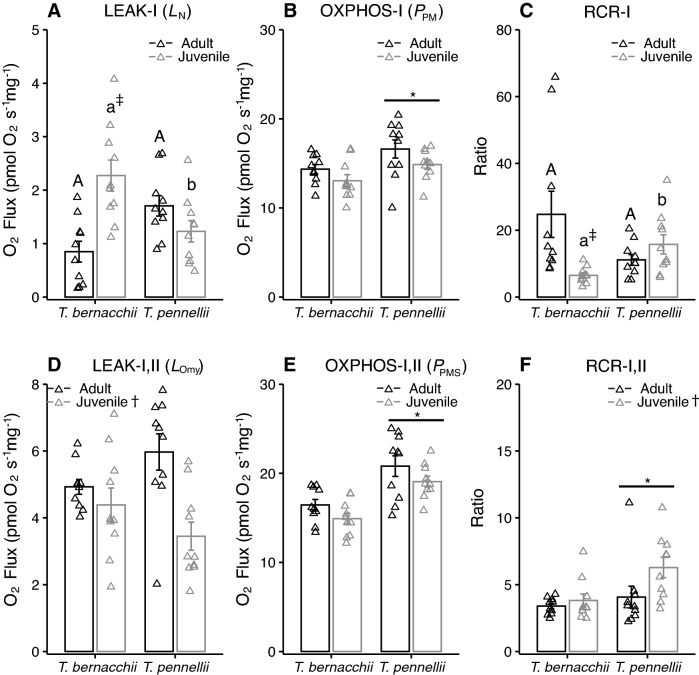
Mitochondrial respiration during LEAK and OXPHOS states in permeabilized ventricle fibers of juvenile and adult *Trematomus bernacchii* and *T. pennellii*. There was a significant interaction of species and life history stage on **A** LEAK-I respiration (*L*_N_; two-way ANOVA; species: *F*_*(*1,36)_ = 0.5, *P* = 0.96, life history stage: *F*_(1,36)_ = 3.5, *P* = 0.14, species X life history stage: *F*_(1,36)_ = 16.3, *P* < 0.01). There was a significant effect of species on **B** OXPHOS-I respiration measured with maximal ADP and substrates of complex I (*P*_PM_; two-way ANOVA; species: *F*_(1,36)_ = 8.1, *P* = 0.01, life history stage: *F*_(1,36)_ = 4.5, *P* = 0.08, species X life history stage: *F*_(1,36)_ = 0.1, *P* = 1). There was significant interaction of species and life history stage on **C** respiratory control ratio RCR-I (*P*_PM_/*L*_N_; two-way ANOVA; species: *F*_(1,36)_ = 0.3, *P* = 1, life history stage: *F*_(1,36)_ = 5.2, *P* = 0.06, species X life history stage: *F*_(1,36)_ = 15.4, *P* < 0.01). There was a significant effect of life history stage on **D** LEAK-I,II respiration (*L*_Omy_; two-way ANOVA; species: *F*_(1,36)_ = 0.1, *P* = 1, life history stage: *F*_(1,36)_ = 12.1, *P* < 0.01, species X life history stage: *F*_(1,36)_ = 5.1, *P* = 0.06). There was a significant effect of species on **E** OXPHOS-I,II respiration measured with maximal ADP and substrates of complex I and II (*P*_PMS_; two-way ANOVA; species: *F*_(1,36)_ = 29.4, *P* < 0.01, life history stage: *F*_(1,36)_ = 4.3, *P* = 0.09, species X life history stage: *F*_(1,36)_ = 0.1, *P* = 1) and there was a significant effect of species and life history stage on **F** respiratory control ratio RCR-I,II (*P*_PMS_/*L*_Omy_; two-way ANOVA; species: *F*_(1,36)_ = 6.4, *P* = 0.03, life history stage: F_(1,36)_ = 5.9, P = 0.04, species X life history stage: *F*_(1,36)_ = 3.2, *P* = 0.08). Sample size was 10 individuals for each species and life stage. An asterisk indicates significance between species and a dagger indicates significance between life stages. If an interaction was detected, different upper case letters indicate significant differences between adults, different lower case letters indicate significant differences between juveniles and a double dagger indicates significant difference between life history stages within a species. Data are presented as mean ± SEM

There was significant interaction of species and life history stage on LEAK-I respiration (Fig. 3A). Juvenile *T. bernacchii* had significantly higher LEAK-I respiration than adult *T. bernacchii* and juvenile *T. pennellii*. In contrast, there was significant effect of life history stage in LEAK-I,II respiration, with lower respiration in the juveniles as compared to adults in both species (Fig. 3D). There was a significant effect of species but not life history stage on OXPHOS-I respiration (Fig. 2B) and OXPHOS-I,II (Fig. 2E), with higher OXPHOS respiration in *T. pennellii*. There was a significant interaction of species and developmental stage on RCR-I (Fig. 3C), with a significantly lower RCR-I in juvenile *T. bernacchii* than in adult *T. bernacchii* or juvenile *T. pennellii.* There was significant effects of species and developmental stage on RCR-I,II (Fig. 3F); the effect of high RCR-I,II of juvenile *T. pennellii* was likely driven by low LEAK-I,II respiration.

**Fig. 4:**
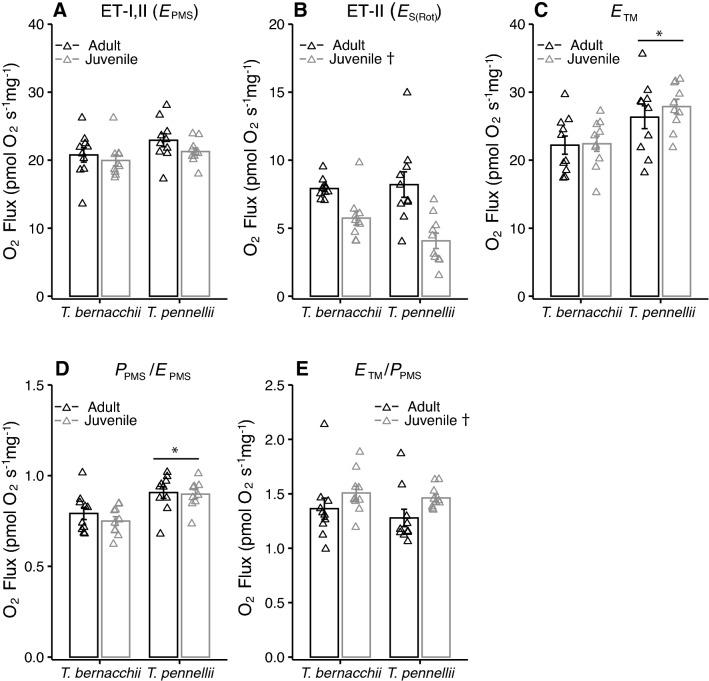
Electron transport capacity in ventricle fibers of juvenile and adult *Trematomus bernacchii* and *T. pennellii*. There was no effect of species or life history stage on **A** electron transport capacity (ET-I, II) measured in mitochondria uncoupled with FCCP using substrates of complex I and II (*E*_PMS_: two-way ANOVA; species: *F*_(1,36)_ = 4.0, *P* = 0.11, life history stage: *F*_(1,36)_ = 2.1, *P* = 0.32, species X life history stage: *F*_(1,36)_ = 0.2, *P* = 1). There was an effect of life history stage on **B** ET-II through complex II (*E*_S(Rot)_; two-way ANOVA; species: *F*_(1,36)_ = 1.3, *P* = 0.54, life history stage: F_(1,36)_ = 26.2, *P* < 0.01, species X life history stage: *F*_(1,36)_ = 2.5, *P* = 0.25). There was a significant effect of species on **C** complex IV respiration stimulated by TMPD and ascorbate (*E*_TM_; two-way ANOVA; species: *F*_(1,36)_ = 13.0, *P* < 0.01, life history stage: F_(1,36)_ = 0.4, *P* = 1, species X life history stage: *F*_(1,36)_ = 0.3, *P* = 1), and **D** the phosphorylation pathway control ratio (*P*_PMS_/*E*_PMS_; two-way ANOVA; species: *F*_(1,36)_ = 20.9, *P* < 0.01, life history stage: *F*_(1,36)_ = 0.7, *P* = 0.77, species X life history stage: *F*_(1,36)_ = 0.3, *P* = 1). There was a significant effect of life history stage on **E** excess complex IV capacity calculated as the ratio of complex IV to OXPHOS respiration (*E*_TM_/*P*_PMS_; two-way ANOVA; species: *F*_(1,36)_ = 0.8, *P* = 0.74, life history stage: *F*_(1,36)_ = 6.2, *P* = 0.03, species X life history stage: *F*_(1,36)_ = 0.1, *P* = 1). Sample size was 10 individuals for each species and life stage. An asterisk indicates significance between species and a dagger indicates significance between life stages. Data are presented as mean ± SEM

Heart ET capacity through complex I and II did not differ among juveniles and adults of *T. bernacchii* and *T. pennelllii* (Fig. 4A); however, there was a significant effect of life stage on ET capacity through complex II only, with juveniles of both species exhibiting lower O_2_ flux than the adults (Fig. 4B). There was an effect of species on complex IV respiration, with greater respiration by *T. pennellii* than *T. bernacchii*, (Fig. 4C), while there was a significant effect of life history stage only on excess complex IV respiration, where the juvenile stage of both species showed greater excess complex IV respiration than the adult stage (Fig. 4E). There was an effect of species on the phosphorylation pathway control ratio, with *T. pennellii* exhibiting a higher ratio than *T. bernacchii* when both juvenile and adult stages were taken into account (Fig. 4D).

### Mitochondrial enzyme activities

**Fig. 5:**
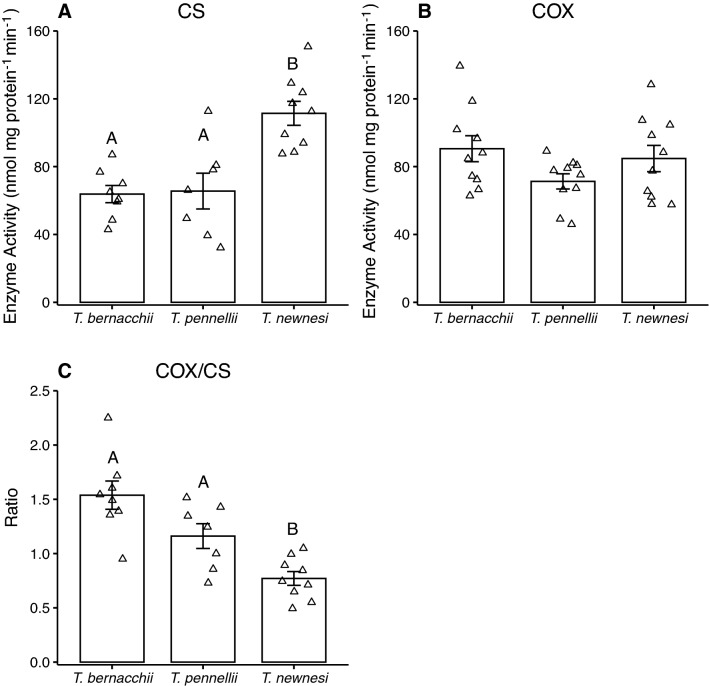
Maximal enzyme activity of citrate synthase (CS) and cytochrome c oxidase (COX) in ventricle of adult *Trematomus bernacchii*, *T. pennellii* and *T. newnesi*. There was a significant effect of species on **A** CS activity (ANOVA; *F*_(2,21)_ = 13.3, *P* < 0.01). There was no effect of species on **B** COX activity (ANOVA; *F*_(2,27)_ = 2.1, *P* = 0.28). There was an effect of species on **C** the ratio of COX to CS activity (ANOVA; F_(2,21)_ = 14.8, *P* < 0.01). Sample size was 8 for adult *T. bernacchii*, 7 for adult *T. pennellii* and 9 for adult *T. newnesi* for CS activity. Sample size was 10 individuals for each species and life stage for COX activity. Different letters indicate significant differences among species. Data are presented as mean ± SEM

Maximal enzyme activity of CS was significantly higher in the heart of adult *T. newnesi* than either *T. bernacchii* or *T. pennellii* (Fig. 5A), while there was no difference in COX activity of the heart among the three species (Fig. 5B). The ratio of COX to CS differed among the species, with the ratio significantly lower in *T. newnesi* than the other two notothenioids (Fig. 5C).

**Fig. 6:**
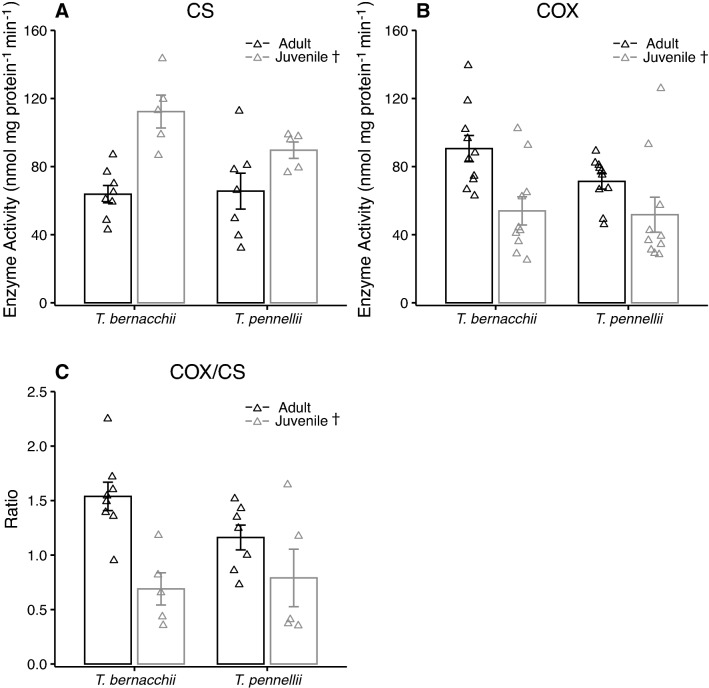
Maximal enzyme activity of citrate synthase (CS) and cytochrome c oxidase (COX) in ventricle of juvenile and adult *Trematomus bernacchii* and *T. pennellii*. There was a significant effect of life history stage on **A** CS activity (two-way ANOVA; species: *F*_(1,21)_ = 0.7, *P* = 0.80, life history stage: *F*_(1,21)_ = 20.0, *P* < 0.01, species X life history stage: *F*_(1,21)_ = 2.2, *P* = 0.30), **B** COX activity (two-way ANOVA; species: *F*_(1,36)_ = 1.6, *P* = 0.44, life history stage: *F*_(1,36)_ = 17.6, *P* < 0.01, species X life history stage: *F*_(1,36)_ = 0.4, *P* = 1) and **C** the ratio of COX to CS activity (two-way ANOVA; species: *F*_(1,21)_ = 1.7, *P* = 0.42, life history stage: *F*_(1,21)_ = 14.4, *P* < 0.01, species X life history stage: *F*_(1,21)_ = 2.2, *P* = 0.31). Sample size was 8 for adult *T. bernacchii* and 7 for adult *T. pennellii* and 5 for juveniles of *T. bernacchii and T. pennellii* for CS activity. Sample size was 10 individuals for each species and life stage for COX activity. A dagger indicates significance between life stages. Data are presented as mean ± SEM

Juveniles of *T. bernacchii* and *T. pennellii* had significantly higher heart CS activity than the adult counterparts (Fig. 6A). In contrast, heart COX activity was lower in the juveniles than the adults of *T. bernacchii* and *T. pennellii* (Fig. 6B). The ratio of COX to CS in the heart was lower in the juveniles than the adults (Fig. 6C). There was no effect of species on either CS or COX heart activity.

## Discussion

Impacts on an organism’s energetic equilibrium can occur with variations in temperature, salinity, O_2_ and CO_2_, which are some of the environmental parameters in Antarctic waters that are predicted to fluctuate as a result of climate change (Dierssen et al. 2002; McNeil et al. 2010; Schmidtko et al. 2017). How well organisms cope as the environment transitions from a long historic period of stability to one of change, will depend on their metabolic capacity; a complex physiological trait, significantly influenced by mitochondrial function. Understanding baseline variation in mitochondrial physiology among species occupying different ecological roles can provide important clues to mechanistic underpinnings of species aerobic performance that could be a significant determinant of whole animal performance. In the present study, species differences were evident in adult notothens from the subfamily Trematominae across the different mitochondrial respiratory states of permeabilized fibers, indicating variation in aerobic capacity of the cardiac tissue. Generally, the pelagic *Trematomus newnesi* exhibited the highest capacity of mitochondrial respiratory flux (i.e. OXPHOS) and mitochondrial efficiency (i.e. RCR) as compared to the benthic species *T. bernacchii* and *T. pennellii*. Greater mitochondrial content, as quantified by CS activity, was found in *T. newnesi*, and is likely the mechanism that accounts for the increased OXPHOS capacity of cardiac tissue. Species differences between *T. bernacchii* and *T. pennellii* were evident when mitochondrial function of juvenile and adult stages were compared together, such that greater OXPHOS respiration was found in *T. pennellii*. Life stage largely had an effect on mitochondrial efficiency and excess complex IV capacity, but little effect on OXPHOS respiration and ET capacity. Metabolic capacities of cardiac tissue differed between the species, which may be linked to their distinct ecological roles, and may offer different constraints on acclimation capacity to changing ocean conditions.

### Coupled state respiration in adult notothenioids

LEAK respiration is the resting state of mitochondria, with the O_2_ flux compensating for proton leak, proton slip, cation cycling and electron leak, and thus not contributing to the biochemical work and the production of ATP (Pesta and Gnaiger 2011). While there was some species variation in LEAK respiration measured through complex I, this was compensated by higher OXPHOS flux, which resulted in no significant difference among the three species in mitochondrial efficiency of the mitochondria. When both complex I and II were considered in tandem, however, mitochondrial efficiency was the highest in *T. newnesi*, as a result of low LEAK respiration with simultaneously high OXPHOS respiration measured through complex I and II. LEAK respiration and in turn mitochondrial efficiency may be altered in response to changing environmental conditions such as warming and a rise in *P*CO_2_. An increase in temperature in notothenioid species such as *Lepidonotothen nudifron* (Hardewig et al. 1999), *Pachycara brachycephalum* (Lanning et al. 2005) and *Chaenocephalus aceratus* (O’Brien et al. 2018), and an increase in *P*CO_2_ in *Notothenia rossii* (Strobel et al. 2012) has been shown to induce uncoupling leading to decreased efficiency of mitochondrial oxidation. In contrast, mitochondrial efficiency remained constant in *T. bernacchii* up to 18 °C (Weinstein and Somero 1998). Therefore, while baseline differences in LEAK respiration and mitochondrial efficiency may contribute to differences in aerobic performance in the closely related notothenioids of this study, changes in these mitochondrial parameters in response to changes in environmental conditions, may be species-specific and will require additional experiments.

The respiratory capacities for OXPHOS-I and OXPHOS-I,II in the cardiac muscle was significantly higher in *T. newnesi* than either *T. bernacchii* or *T. pennellii*. Enhanced capacity for mitochondrial respiratory flux can facilitate mitochondrial respiration during periods of environmental stress, suggesting that *T. newnesi* has potential for greater mitochondrial function under stressful conditions than the other two notothenioids. Population or species differences in OXPHOS respiratory flux have previously been linked to variation in the environmental conditions, with higher respiratory capacities in species experiencing greater intensity of environmental stress. As examples, highland populations of torrent ducks (*Merganetta armata*) and deer mice (*Peromuscus maniculatus*) were found to have higher respiratory capacity in the gastrocnemius muscle than their lowland counterparts, likely an important factor for enhanced performance in cold, hypoxic conditions (Dawson et al. 2016; Mahalingam et al. 2017 respectively). In New Zealand triplefin fishes, the intertidal *Bellapiscis medius*, a species that is more hypoxia and temperature tolerant, had greater cardiac OXPHOS capacity than the two subtidal species, *Forsterygion varium* and *Forsterygion malcolmi* (Hilton et al. 2010). These studies demonstrate that enhanced mitochondrial respiratory capacity can be linked to greater whole animal performance in organisms inhabiting more variable, stressful environments. In the case of the notothenioids, which have evolved under stable environmental conditions for the past several million years, species differences in mitochondrial respiratory capacity cannot be attributed to variation in environmental factors such as temperature, oxygen or salinity. It might be, however, that *T. newnesi*, with a greater respiratory capacity for OXPHOS, is better poised than *T. bernacchii* or *T. pennellii* at maintaining aerobic energy supply if there is an increase in energy demand, resulting from environmental disturbance.

Complex IV in vitro O_2_ flux rates are often greater than that of maximal ADP-stimulated OXPHOS, with this excess capacity of complex IV particularly evident in aerobic tissues, such as the heart (Gnaiger et al. 1998). Consistent with this, all three notothenioid fishes had excess complex IV capacity in cardiac tissue, with *T. newnesi* exhibiting significantly greater excess capacity than either T*. bernacchii* or *T. pennellii*. Despite complex IV generally being in excess capacity, it is considered to be the rate-limiting step in the electron transport system and has been shown to have a significant control over mitochondrial respiration rates (Arnold 2012). The excess complex IV capacity is thought to be important in maintaining the electron transport system in an oxidized state, keeping favorable thermodynamic gradient under most conditions (Blier and Lemieux 2001) or alternatively it has been thought to be a necessary regulatory mechanism for maintaining a high O_2_ affinity by a low complex IV turnover rate (Gnaiger et al. 1998). Greater excess complex IV capacity has been linked to enhanced mitochondrial function and hypoxia performance in highland torrent ducks (Dawson et al. 2016) and New Zealand triplefin fish *Bellapiscis medius* (Hilton et al. 2010). In notothenioid fishes, greater cardiac performance in *Notothenia coriiceps* in relation to *Chaenocephalus aceratus*, has been at least partly attributed to excess complex IV capacity (O’Brien et al. 2018). In the current study, while all three notothenioid species show excess complex IV capacity, in T*. newnesi*, greater excess complex IV capacity may contribute to enhanced aerobic performance over a broader range of conditions than in *T. bernacchii* and *T. pennellii*.

Species belonging to subfamily Trematominae, which include *T. bernacchii*, *T. pennellii* and *T. newnesi*, occupy different ecological niches, ranging from benthic, pelagic to cryopelagic (Van de Putte et al. 2012). Both *T. bernacchii* and *T. pennellii* are less active, benthic species (Gon and Heemstra 1990), while *T. newnesi* is classified as an active, semi-pelagic (Van de Putte et al. 2012) or even cryopelagic species inhabiting the underside of the sea ice (La Mesa et al. 2000; Eastman and DeVries 1997). A difference in OXPHOS capacity between pelagic and benthic Notothenioid fishes of the Antarctic Peninsula has previously been documented, with OXPHOS respiration of the pelagic species *Pleuragramma antarcticum* twice that of its benthic counterparts (Martinez et al. 2013). Similarly, in this study, the pelagic species *T. newnesi*, was found to exhibit significantly higher complex I-linked and complex I and II-linked OXPHOS respiration than either of the benthic notothenioids. Taking into account both studies, there appears to be a correlation between mitochondrial phenotype and species ecotype in notothenioid fishes; active, pelagic species have greater mitochondrial respiratory capacity than the less active, benthic species. This variation in mitochondrial capacity may underlie variation in aerobic metabolism at the whole animal level and is not surprising given the different ecological and locomotory demands of the species (Todgham and Mandic 2020).

Variation in respiratory flux of permeabilized fibers among the species represents tissue-level difference that may be a result of intrinsic species differences of the catalytic capacity of the mitochondrial components or a difference in mitochondrial density. In this study, the pelagic *T. newnesi* had the highest CS activity, indicating greater mitochondrial density as compared to the two benthic notothenioids, implicating that the greater OXPHOS respiratory flux of permeabilized fibers in *T. newnesi* was likely due to significantly more mitochondria in the heart. Furthermore, *T. newnesi* had similar COX activity to the other two species, that when standardized to CS activity (COX to CS ratio), indicated less COX per mitochondria and, therefore, a lower capacity at the level of the mitochondria. High mitochondrial density appears to be an important driver of tissue level difference in respiration capacity between the species. Other studies in notothenioid fishes found that the variation in respiratory flux capacity between benthic and pelagic fishes was not attributed to differences in mitochondrial density (Martinez et al. 2013). Likewise there was no consistent difference in mitochondrial density between active, pelagic species and relatively sedentary, demersal species of Antarctic and sub-Antarctic notothenioids (Johnston et al. 1998). However, similar to the current study, in temperate fishes, a two to threefold higher mitochondrial respiration in the pelagic as compared to benthic species was mainly due to differences in mitochondrial volume density (Burpee et al. 2010).

### Uncoupled state respiration in adult notothenioids

Following the same pattern as OXPHOS capacity, *T. newnesi* had significantly higher ET capacity than *T. bernacchii* and *T. pennellii*. In all three species ET capacity was higher than OXPHOS capacity, as evidenced by the flux control ratio of the respiratory capacities for OXPHOS relative to electron transport (*P*/*E*) to be less than 1. This flux control ratio provides an indication of the control of the phosphorylation system (i.e., ATP synthase, adenine nucleotide translocase and inorganic phosphate transporters) on mitochondrial respiration (Gnaiger 2009; Pesta and Gnaiger, 2011), which can vary across species (Du et al. 2017). While there was some variation among the notothenioids tested in this study, the phosphorylation system had a restraining influence on OXPHOS respiration in the heart of all three species.

### Mitochondrial respiration in adult versus juvenile notothenioids

Early life stages are typically more vulnerable to environmental fluctuations than adult fish (Pankhurst and Munday 2011), with differences between stages across ontogeny. A combination of exposure to warming and ocean acidification during early development caused a significant decrease in survival and developmental rate in the Antarctic dragonfish embryos (*Gymnodraco acuticeps*, Flynn et al. 2015), while 9 weeks of acclimation at 4 °C for the juvenile *T. bernacchii* incurred a substantial metabolic cost as seen by an 84% reduction of mass growth (Sandersfeld et al. 2015). Mitochondrial function and in turn aerobic performance may be lower in early life stages as compared to adults, contributing to the sensitivity of the early life stages to environmental variability. Contrary to this prediction, however, mitochondrial function of juvenile fish was not found to be lower than that of the adult fish and aspects of mitochondrial function such as mitochondrial efficiency and excess complex IV capacity of the permeabilized fibers were found to be greater in the juvenile fish. There was a significant effect of life stage on mitochondrial efficiency in *T. bernacchii* and *T. pennellii*, where juvenile fish exhibited greater mitochondrial efficiency than the adult fish. High efficiency as measured by flux through complex I and II was particularly evident in juvenile *T. pennellii*, and appears to be driven by low LEAK respiration rather than high OXPHOS respiratory capacity. Indeed, there was no significant difference between juvenile and adult individuals with regard to respiratory capacity for OXPHOS in the permeabilized heart fibers. Despite a lack of difference in OXPHOS capacity, juvenile individuals possessed greater excess complex IV capacity than adult fish. While not well understood, one hypothesis is that the high complex IV capacity is required to prevent excessive rise of intracellular O_2_ concentration and generation of reactive O_2_ species (ROS; Papa et al. 1997). Differences in the antioxidant defense system in the notothenioids *N. rossii* and *N. coriiceps* were found to be related to their ecological niches; the more active, benthopelagic *N. rossii*, were found to have a greater antioxidant defense system than the sedentary, benthic *N. coriiceps* (Klein et al. 2017). Similarly, the cryopelagic juvenile fish in this study are more active than the benthic adults and as such the greater excess complex IV capacity may be a defense mechanism, preventing the higher accumulation of ROS.

Heart COX activity and COX activity standardized to CS was significantly lower in juveniles of *T. bernacchii* and *T. pennellii*, indicating that at a per mitochondria level ETS proteins were less dense and mitochondrial capacity of juveniles was lower than that of the adults. Activity of CS, however, was significantly greater in juveniles, pointing to greater mitochondrial density than the adults. High mitochondrial density in juveniles compensated for lower mitochondrial capacity and resulted in similar OXPHOS and greater excess complex IV capacity at the heart tissue level.

## Conclusions

Respiratory flux capacity of the intact mitochondria of permeabilized fibers differed among the adult notothenioids in this study occupying different ecological niches, indicating species differences in metabolic capacity of the heart. Different metabolic capacities point to variation in acclimation potential among the species and a differential sensitivity to environmental change, with *T. newnesi* potentially better poised to compensate for the predicted changing ocean conditions. This is, however, dependent on how much of the metabolic capacity is currently in excess that would be available to restore energetic equilibrium during periods of environmental stress. In the current study, species differences are based on data from a single population found in McMurdo Sound, which may be a limitation if, within a species, there are population level differences in metabolic capacity. For example, it is possible that populations of *T. bernacchii* inhabiting the more thermally variable Western Antarctic Peninsula may possess greater metabolic capacity and acclimation potential then populations located in McMurdo Sound, setting up important comparative population work that would better inform species-level response to environmental change.

In juveniles, metabolic capacity of the heart was fairly consistent between the life history stages. Recent evidence suggests that juvenile *T. bernacchii*, in their second year of age, were not significantly different from adults with regards to the time-frame for complete temperature compensation of RMR during exposure to warming (Davis et al. 2018). Therefore, it is possible that older juvenile Antarctic fish are not at any greater susceptibility to future climate change effects than the adults. High environmental vulnerability may still occur at younger life stages, particularly in the embryos (e.g. Flynn et al. 2015) and the larvae, and as such, it is important to understand mitochondrial function across early development to better pinpoint the most vulnerable stage during ontogeny. This is logistically challenging due to difficulty in capturing and conducting research on the early life stages and will entail committing resources in identifying the Antarctic notothenioids for which this type of research may be feasible.
